# Treatment of severe neutropenia with high-dose pyridoxine in a patient with chronic graft versus host disease and squamous cell carcinoma: a case report

**DOI:** 10.1186/1752-1947-5-372

**Published:** 2011-08-12

**Authors:** Mariam Rauf, Charise Gleason, Ajay K Nooka, Abbie Husman, Edmund K Waller

**Affiliations:** 1 Dow Medical College, Dow University of Health Sciences, A-167, Block 13-C, Gulshan-e-Iqbal; Karachi, Pakistan; 2 Winship Cancer Institute, Emory University School of Medicine, 365B Clifton Road NE, Room B5119, Atlanta, GA 30322, USA

## Abstract

**Introduction:**

The differential diagnosis of neutropenia includes medications, infections, autoimmune diseases, and deficiencies of Vitamin B12 and folate. The association of Vitamin B6 deficiency with severe neutropenia is a rare finding.

**Case presentation:**

A 51-year-old Caucasian woman presented with fever and profound neutropenia (48 neutrophils/uL). Her clinical history included non-Hodgkin lymphoma, in remission following treatment with allogeneic bone marrow transplantation, quiescent chronic graft-versus-host disease, and squamous cell carcinoma of the skin metastatic to cervical lymph nodes. Medications included atenolol, topical clobetasol, Ditropan (oxybutynin), prophylactic voriconazole, prophylactic valganciclovir, Soriatane (acitretin), and Carac (fluorouracil) cream. The bone marrow was hypocellular without metastatic cancer or myelodysplasia. Neutropenia did not respond to stopping medications that have been associated with neutropenia (valganciclovir, voriconazole and Soriatane) or treatment with antibiotics or granulocyte colony stimulating factor. Blood tests revealed absence of antineutrophil antibodies, normal folate and B12 levels, moderate zinc deficiency and severe Vitamin B6 deficiency. Replacement therapy with oral Vitamin B6 restored blood vitamin levels to the normal range and corrected the neutropenia. Her cervical adenopathy regressed clinically and became negative on scintography following Vitamin B6 therapy and normalization of the blood neutrophil count.

**Conclusion:**

Severe pyridoxine deficiency can lead to neutropenia. Screening for Vitamin B6 deficiency, along with folate and Vitamin B12 levels, is recommended in patients with refractory neutropenia, especially those with possible malabsorption syndromes, or a history of chronic-graft-versus host disease. Severe neutropenia may facilitate progression of squamous cell carcinoma.

## Introduction

Neutropenia has been associated with medications, infections, autoimmune diseases, and deficiencies of Vitamin B12 and folate [1]. An association of Vitamin B6 deficiency with severe neutropenia is a rare finding [2-4]. We describe the case of a patient who had severe neutropenia (neutrophil count of 48 cells/uL) associated with profound Vitamin B6 deficiency that markedly improved with high dose Vitamin B6 therapy.

## Case Presentation

**Figure 1:**
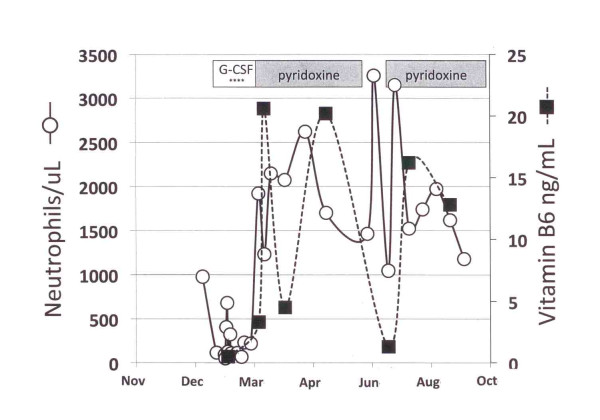
**Resolution of neutropenia and Vitamin B6 deficiency following oral pyridoxine supplementation**. The absolute neutrophils per microliter blood are shown in open circles with a solid line. The blood levels of Vitamin B6 are shown as filled squares, with the dashed line. The duration of pyridoxine administration (150 mg/day) is shown as the horizontal grey bars (with a brief period of non-compliance in July). The administration of 300 ug sub-cutaneous G-CSF every other day is shown with asterisks.

The patient was a 51-year-old Caucasian woman with relapsed non-Hodgkin lymphoma treated four years earlier with an allogeneic stem cell transplant. Her lymphoma was in remission. Complications of her allogeneic transplant included a history of chronic extensive graft-versus-host disease (GvHD) involving the skin and dermal squamous cell carcinoma. Immunosuppression with cyclosporine had been discontinued two months earlier due to the development of squamous cell carcinoma and the absence of active GvHD. She had no history of smoking, alcohol, or drug abuse. She presented with fever and profound neutropenia; serial blood counts showed that the neutropenia had been present for more than a month (Figure 1). Her examination showed moderate cachexia, long-standing sclerodermatous changes and hyper-pigmentation of the skin consistent with quiescent GvHD, palpable 2.5 cm left cervical and submandibular adenopathy that was flurodeoxy glucose (FDG) avid on positron emission tomography/computerized tomography (PET/CT) scan. She had a non-focal neurological examination. A complete blood count showed a normal total leukocyte count (4,600/uL) with 2% granulocytes (48 neutrophils/uL) together with 84% lymphocytes and 14% monocytes. Red blood cells were 4.24 × 10E12/L, hemoglobin was 12.4 g/dL, hematocrit was 0.37, and the platelet count was 113 × 10E9/L. Medications included atenolol, topical clobetasol, Ditropan (oxybutynin), prophylactic voriconazole, prophylactic valganciclovir, Soriatane (acitretin), and Carac (fluorouracil) cream. Blood cultures showed a micrococcus species. Her iron level was 330 mcg/L with 20% saturation. Folate, vitamin B1, vitamin B12, copper, vitamin D levels, thyroid function tests, creatinine, bilirubin, liver enzymes and lactate dehydrogenase (LDH) were normal. Her blood zinc level was borderline low at 49 mcg/dL. Antineutrophil antibodies were absent (<1:10 titer). PCR tests for cytomegalovirus (CMV) and Epstein Barr virus (EBV) in the blood were negative, and had remained negative on prophylactic doses of valganciclovir following CMV reactivation five months earlier. Her vitamin B6 level was very low at 0.5 ng/mL (reference range 5 to 30 ng/mL). Serum IgG was polyclonally elevated at 3720 mg/dL as were blood T-cells (4580 cells/uL) and CD8+ T-cells (2836 cells/uL) with normal numbers of CD4+ T-cells, B-cells and natural killer (NK) cells.

Her fever resolved with a course of vancomycin, without identifying a site of infection. Histological examination of the marrow demonstrated marked hypocellularity without specific megaloblastic or myelodysplastic findings, metastatic cancer, or features to suggest immune-mediated thrombocytopenia. Cytogenetic analysis showed 100% donor (male) cells without any clonotypic abnormalities. Biopsy of a cervical lymph node showed poorly differentiated carcinoma, consistent with metastatic squamous cell carcinoma. Valganciclovir, voriconazole, and Soriatane (acitretin) were discontinued (as potential causes of neutropenia) and prophylactic fluconazole and acyclovir were started. Granulocyte colony stimulating factor (G-CSF; 300 ug subcutaneously every other day) was administered for four doses in mid February 2010 without any improvement in her neutropenia. Following daily supplementation with 150 mg Vitamin B6, blood B6 levels normalized concomitant with resolution of the neutropenia (Figure 1). Following correction of the B6 deficiency and neutropenia, the cervical adenopathy completely regressed by clinical examination and became scintographically negative by PET/CT.

## Discussion

The differential diagnosis for neutropenia in our patient includes drug effects, infection, the immune-suppressive effects of chronic GvHD, autoimmunity [5], and a deficiency of micronutrients [2-4]. Her neutropenia failed to respond to discontinuation of drugs that have been associated with neutropenia, was not associated with recent CMV viremia or antineutrophil antibodies, and was not associated with sepsis or a flare of her chronic GvHD. Based on the clinical history of a rapid response of neutropenia to high-dose Vitamin B6 replacement therapy and a review of literature, we propose that this case of neutropenia is most consistent with a deficiency of Vitamin B6. Vitamin B6 treatment has been reported to be effective in refractory neutropenia due to chronic benzene, aspirin, sulfathiazole, and thiouracil exposure [2-4]. The typical manifestations of Vitamin B6 deficiency [6], including seborrhea dermatitis, intertrigo, atrophic glossitis, angular chelitis, conjunctivitis, and neurologic symptoms were not noted in our patient, although depression was diagnosed and treated around the same time as neutropenia was noted. She also had moderate thrombocytopenia. A micronutrient deficiency may also have contributed to her thrombocytopenia, although thrombocytopenia is common in patients with extensive chronic GvHD. The cause of her profound Vitamin B6 deficiency and moderate zinc deficiency was likely due to malabsorption due to the persistent effects of the prior chronic GvHD.

Squamous cell carcinoma is a known complication of cutaneous chronic GvHD and has been associated with voriconazole therapy in patients receiving chronic immune-suppressive drug therapy [7]. The regression of squamous cell carcinoma that had metastasized to the cervical lymph nodes following correction of neutropenia may reflect the effect of stopping voriconazole and immuno-suppressive drug therapy and/or the role of innate immune responses to this cancer.

## Conclusion

Vitamin B6 deficiency can be associated with neutropenia. Screening for Vitamin B6 deficiency, along with folate and vitamin B12 levels, is recommended in patients with refractory neutropenia, especially those with malabsorption syndromes, history of chronic GvHD, or history of chronic benzene exposure.

## Abbreviations

CMV: cytomegalovirus; EBV: Epstein Barr virus; FDG: fluro-deoxy glucose; G-CSF: granulocyte colony stimulating factor; GvHD: graft-versus-host disease; NK: natural killer cells; PET/CT: positron emission tomography/computerized tomography.

## Consent

Written informed consent was obtained from the patient for publication of this case report and accompanying clinical data. A copy of the written consent is available for review by the Editor-in-Chief of this journal.

## Competing interests

The authors declare that they have no competing interests.

## Authors' contributions

MR analyzed and interpreted the patient data regarding the neutropenia and Vitamin B6 deficiency and wrote the manuscript. CH and AN examined the patient, summarized the medical history, and edited the manuscript. AH performed the histological examination of the marrow. EKW analyzed the data, drew the figure, edited and wrote the final version of the manuscript. All authors read and approved the final manuscript.
